# Leprosy Mimicking Thrombangiitis Obliterans (Buerger’s Disease): A Case Study

**DOI:** 10.7759/cureus.80822

**Published:** 2025-03-19

**Authors:** Caio O Sena, Isabela Maria B Goulart, Pâmella C Justino Sena, Juliana C Justino Omar, Bruno C Dornelas

**Affiliations:** 1 Department of Pathology, University of Uberlândia Clinical Hospital/Empresa Brasileira de Serviços Hospitalares (EBSERH), Uberlândia, BRA; 2 National Reference Center for Sanitary Dermatology and Leprosy, University of Uberlândia Clinical Hospital/Empresa Brasileira de Serviços Hospitalares (EBSERH), Uberlândia, BRA; 3 Department of Ophthalmology, University of Uberlândia Clinical Hospital/Empresa Brasileira de Serviços Hospitalares (EBSERH), Uberlândia, BRA

**Keywords:** borderline lepromatous leprosy, buerger's disease, mycobacterium leprae, thromboangiitis obliterans, vasculitis

## Abstract

Thromboangiitis obliterans (TAO), also known as Buerger’s disease, is a vasculitis associated with a history of smoking, presenting as limb ischemia. Conversely, leprosy is a chronic infectious disease caused by *Mycobacterium leprae* (*M. leprae*). While leprosy typically presents with neurological signs, this report describes a rare case of atypical borderline lepromatous (BL) leprosy in a type 1 reaction with an initial presentation mimicking TAO in an elderly woman. This atypical presentation, combined with skin changes related to aging, masked the diagnosis of BL leprosy.

## Introduction

Thromboangiitis obliterans (TAO), also known as Buerger’s disease, is a vasculitis clinically characterized by limb ischemia of the upper or lower extremities. The differential diagnoses of TAO include cardioembolic and thromboembolic events, vasoconstrictor drug abuse, purpura fulminans, and other vasculitis [1-3]. Conversely, leprosy, or Hansen's disease, is an infectious condition prevalent in tropical regions such as Brazil and India. The etiologic agents of leprosy are acid-fast bacilli (AFB), *Mycobacterium leprae* (*M. leprae*), and *Mycobacterium lepromatosis* (*M. lepromatosis*), which exhibit tropism for macrophages and cells of Schwann, with the ability to infect several other cell types [4,5]. Leprosy presents a wide variety of clinical and histopathological manifestations, which can mimic a broad spectrum of diseases, ranging from localized skin infections, such as tinea, to systemic conditions including rheumatic diseases, namely lupus, scleroderma, and vasculitis [5-7]. However, an initial clinical presentation of leprosy mimicking a vasculitis-like pattern, such as limb ischemia, skin ulcers, and toe necrosis, is rarely described in medical literature. We aim to describe a case of borderline lepromatous (BL) leprosy simulating TAO and emphasize the crucial role of skin biopsy in arriving at the correct diagnosis.

## Case presentation

**Figure 1:**
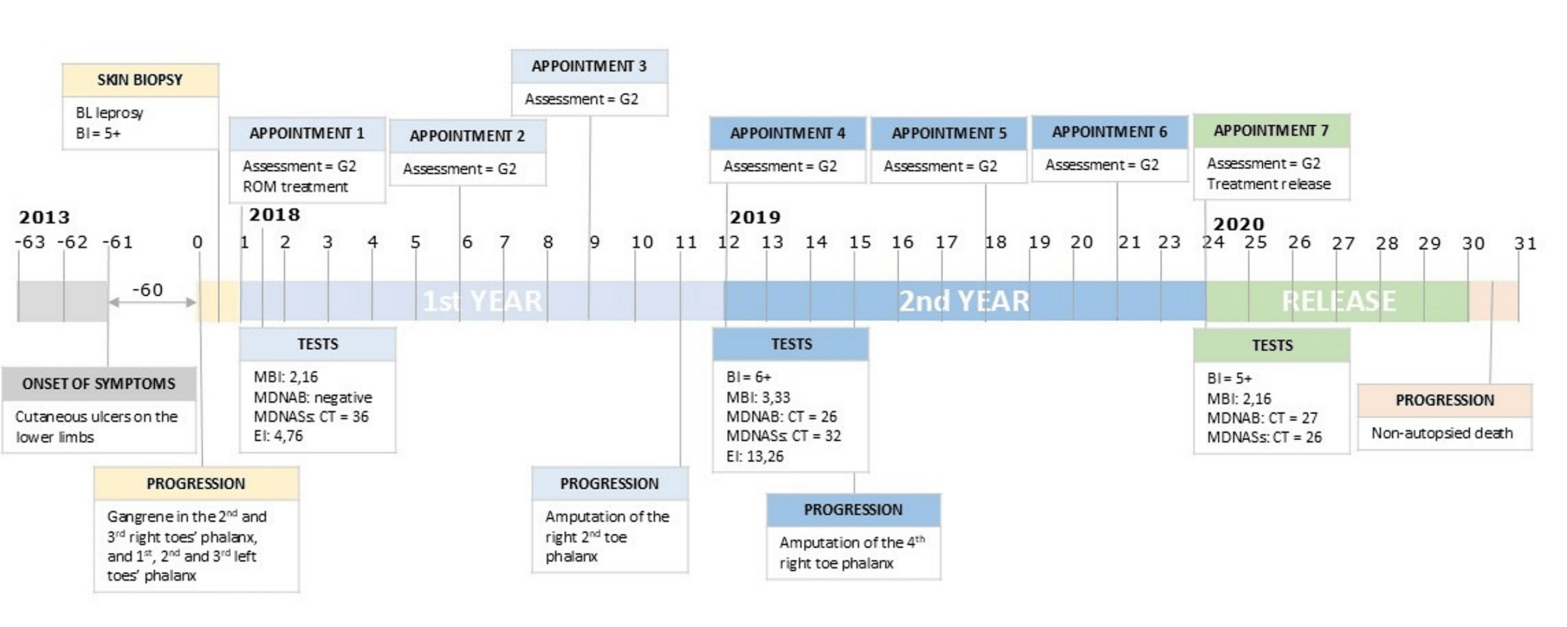
Patient's follow-up over time (scale in months) Month 0 corresponds to the worsening of symptoms that began five years ago. BI: bacillary index in skin biopsy; *Evaluation of physical disability according to the World Health Organization; BL: borderline lepromatous; G2: grade 2 (disability assessment); ROM: rifampicin, ofloxacin, minocycline; CT: cycle threshold. EI: enzyme-linked immunosorbent assay (ELISA) index; MBI: mean bacillary index in skin-slit smear; MDNAB: *Mycobacterium leprae* (*M. leprae*) DNA detection by quantitative polymerase chain reaction (qPCR) in blood; MDNASb: *M. leprae* DNA detection by qPCR in skin biopsy; MDNASs: *M. leprae* DNA detection by qPCR in skin-slit smear.

The patient was an 88-year-old female, a former smoker with complaints of sporadic skin ulcers in the lower limbs for approximately five years (Figure 1).

Her medical history was negative for diabetes mellitus (DM) and systemic arterial hypertension. About a month prior to hospital admission, the patient developed toe necrosis. Upon admission, multiple blackened skin ulcers were observed on both lower limbs. These ulcers had well-defined and slightly hyperemic edges with mild local warmth and were covered by fibrin, with no purulent drainage. Some ulcers had coalesced. Linear skin scars were noticed on the legs, along with dry gangrene of the distal phalanges of the right toes II and III and left toes I, II, and III. The proximal thigh regions were spared. Palpation revealed present and symmetrical femoral, popliteal, anterior tibial, and posterior tibial pulses. Consequently, a clinical diagnosis of TAO was initially formulated.

**Figure 2:**
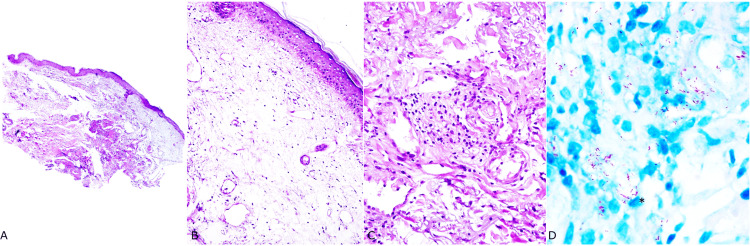
Borderline lepromatous leprosy in a type 1 reaction A) Superficial skin sample with edema and mild mononuclear inflammatory infiltrate (H&E, 4x); B) Marked edema with sparse lymphocytes and foamy macrophages (H&E, 20x); C) In the deep reticular dermis, small aggregates of lymphocytes and foamy macrophages are observed (H&E, 40x); D) Solid and fragmented acid-fast bacilli within histiocytes and endothelial cells (*) (Faraco-Fite, 100x). Image credits: Bruno C. Dornelas and Caio O. Sena

A skin biopsy was performed on the lateral region of the left knee. Microscopic examination showed atrophy of the epidermis and, in the dermis, marked edema with mild, interstitial, perivascular, and deep perineural lymphocytic infiltrate (Figure 2). The presence of foamy macrophages prompted a search for microorganisms. Faraco-Fite staining revealed numerous AFB, both fragmented and solid, within macrophages, endothelial cells, and Schwann cells (bacillary index = 5+). The diagnosis of BL leprosy in a type 1 reaction was established. The patient was subsequently referred to a national reference center for leprosy treatment.

Sensory evaluation using the Semmes-Weinstein monofilament esthesiometer demonstrated loss of deep pressure sensitivity in both feet at all nine points assessed. The initial disability assessment resulted in grade 2 (eyes = 0; hands = 2; feet = 2). At admission, the patient was submitted to laboratory examinations (Table 1).

**Table 1:** Laboratory findings at the hospital and during discharge BUN: blood urea nitrogen; Hct: hematocrit; Hgb: hemoglobin; MCH: mean corpuscular hemoglobin; MCHC: mean corpuscular hemoglobin concentration; MCV: mean corpuscular volume;  RBC: red blood cell count; RDW: red cell distribution width; WBC: white blood cell count

Examination	Result on admission	Result at discharge from hospital	Reference range
Hemogram			
RBC	3.8 million	3.5 million	4.30-5.00 million/µL
Hgb	9.7	9.0	12.0-15.5 g/dL
Hct	29.3	28.4	35%-45%
MCV	77.3	80.9	82-98 fL
MCH	25.6	25.6	26-34 pg
MCHC	33.1	31.7	31-36 g/dL
RDW	15.4	18.1	12%-15 %
WBC	5,200	5,200	3,500-10,500/µL
Neutrophils	3,952	3,848	1,700-8,000 /µL
Lymphocytes	1,144	988	900-2,900/µL
Monocytes	52	260	300-900/µL
Eosinophils	44	52	50-500 /µL
Platelets	300,000	277,000	150,000–450,000/µL
Biochemical tests			
Prothrombin time activity	74	95	70% to 100%
BUN	30.6	36.4	16.6-48.5mg/dL
Creatinine	0.84	0.95	0.5-0.9 mg/dL
Sodium	142.00	140.0	134-145 mEq/L
Potassium	4.36	4.66	3.5-5.5 mEq/L

Skin-slit smears (SSS) taken from seven sites showed a mean bacillary index (MBI) of 4.42. Serology for immunoglobulin M (IgM) anti-phenolic glycolipid-1 (PGL-I) was positive (enzyme-linked immunosorbent assay (ELISA) index = 4.76). Molecular tests revealed negative *M. leprae* DNA detection by real-time quantitative polymerase chain reaction (qPCR) in blood, but positive qPCR in SSS (CT=26; 5.9 x 107 copies/0.5 mL). Considering the hemogram and the potential side effects of dapsone [8] on the hematimetric profile, the patient began a monthly rifampicin, ofloxacin, minocycline (ROM) regimen under supervision, consisting of rifampicin (600 mg), ofloxacin (400 mg), and minocycline (100 mg). Follow-up included laboratory exams at months 12 and 24 per the National Reference Center's protocol. By month 11, the patient developed necrosis of the right second toe, necessitating surgical amputation. In month 14, ischemic necrosis led to the amputation of the right fourth toe. Six months after completing treatment, the National Reference Center was notified of the patient's death from an undetermined cause.

## Discussion

Thromboangiitis obliterans' pathophysiology remains incompletely understood, and its diagnosis primarily relies on a clinical assessment guided by epidemiological factors to approach ischemic limb phenomena [9,10]. Skin biopsy plays a critical role in excluding differential diagnoses of limb ischemia causes [7]. Based on clinical history and physical examination findings, the medical team initially considered Buerger's disease. Despite the patient’s clinical features not aligning with typical TAO demographics (male smokers under 50 years), it was recognized that TAO can affect women over 50 years [11]. Skin biopsy serves as a diagnostic tool in cases involving large artery damage, individuals over 50 years, DM, positive antinuclear antibodies, high anticardiolipin antibodies, subcutaneous nodules, or superficial thrombophlebitis [12]. Microscopic changes in TAO vary across clinical stages: the acute phase reveals an inflammatory occlusive thrombus composed of polymorphonuclear leukocytes, multinucleated giant cells, and microabscesses [10]; during the intermediate phase, inflammatory thrombus progresses to organization, and in the chronic phase, the inflammatory response regresses and thrombus fibrosis [10,12]. However, our case did not exhibit signs of vasculitis in the skin biopsy.

Leprosy’s pathophysiology is comparatively better understood and directly reflects variation in host immune responses to mycobacterial infection. In the lepromatous spectrum, the high bacillary load and abundant PGL-I and lipoarabinomannan (LAM) antigens suppress macrophage activity, facilitating bacterial survival, replication, and disease progression [13-15]. The lepromatous spectrum exhibits an anergic immune response characterized by the predominance of Th2 lymphocytes and regulatory T cells (Treg) producing interleukin 10 (IL-10) and transforming growth factor-beta (TGF-β), resulting in progressive and diffuse skin infiltration, particularly in facial and limb areas, giving rise to a parchment-like, xerotic appearance, in addition to thinning hair on limbs, eyelashes, and eyebrows; it is also common [13-17].

Leprosy reactions manifest as acute inflammatory episodes, categorized into type 1 and type 2. Type 1 leprosy reactions are cell-mediated, while type 2 reactions involve immune complex formation and can lead to conditions like erythema multiforme, erythema nodosum leprosum, Lucio's phenomenon (necrotizing erythema), and neuritis, alongside systemic manifestations [14,16]. However, in the case presented, the histopathological findings are more characteristic of type 1 leprosy reaction. Moreover, despite the absence of constitutional symptoms, necrotic ulcers on the lower limbs and necrosis of the phalanges suggest bacillary involvement in vessel walls and endothelial cells, potentially triggering vasculitis-like events, thrombus formation, and subsequent vessel occlusion leading to ischemia and infarction [15]. This phenomenon may not have been documented in our case due to inadequate sampling or the time-dependent nature of vasculitis findings.

Furthermore, the involvement of the autonomic nervous system in leprosy impacts cutaneous blood flow regulation, particularly in reducing the temperature in the palms and soles, where sympathetic vasoconstrictor innervation is predominant. The loss of sympathetic vasoconstrictor impulses in arteriovenous anastomoses of glabrous skin predisposes to ulcers and gangrene, as observed in our case [18].

Additional factors contributing to leprosy not being initially considered as a primary diagnosis are age-related skin changes such as atrophy of skin appendage and xerosis [19]. Chronic sun exposure can also manifest as wrinkled, parchment-like, and yellowish skin due to elastosis [20]. These factors complicated the identification of cutaneous stigmata in elderly leprosy patients, making leprosy diagnosis a significant public health challenge in Brazil, where 24.1% (19,582/81,205) of new cases diagnosed between 2016 and 2018 occurred in the elderly population [21].

In conclusion, leprosy is typically not considered in the differential diagnosis for TAO, particularly in endemic areas, due to the infrequent initial manifestations resembling vasculitis, such as skin ulcers, ischemia, and necrosis in lower limbs [6,15]. Key diagnostic clues for considering leprosy in such cases include sensory loss, palpable peripheral nerves, and the presence of skin lesions. Clinicians should be vigilant for atypical presentations in elderly patients where age-related skin changes can obscure typical leprosy signs [13-19]. The biopsy of the clinically involved tissue proved crucial for accurate diagnosis and effective treatment of our patient.

## Conclusions

We reported a rare cutaneous manifestation of leprosy mimicking necrotizing vasculitis in an elderly patient to illustrate the broad spectrum of leprosy’s clinical presentation. This case report underscores the importance of broadening the differential diagnosis for TAO to include leprosy, particularly in endemic regions where the disease is prevalent. The atypical presentation of borderline lepromatous leprosy, which can mimic necrotizing vasculitis and lead to significant clinical confusion, emphasizes the need for heightened clinical awareness among healthcare professionals. Factors such as age-related changes in skin physiology and the impact of chronic sun exposure can obscure the classic signs of leprosy, contributing to diagnostic challenges in elderly patients. The critical role of skin biopsy in reaching an accurate diagnosis cannot be overstated, as timely and precise identification of leprosy allows for the initiation of appropriate treatment, ultimately reducing morbidity associated with the disease. This case serves as a reminder of the necessity for vigilance in recognizing diverse manifestations of leprosy to ensure prompt intervention and mitigate potential complications.
